# A Prospective Randomized Double Blind Study to Evaluate the Effect of Infusion of Amino Acid Enriched Solution on Recovery from Neuromuscular Blockade

**Published:** 2009-06

**Authors:** Nishkarsh Gupta, Raminder Sehgal, Rakesh Kumar, Kavita Rani Sharma, Anju Gupta, Nidhi Agrawal

**Affiliations:** 1 Junior Consultant Max, Saket, New Delhi; 2 Senior Consultant, Sir Ganga Ram Hospital, New Rajinder Nagar, New Delhi; 3 Professor, Department of Anaesthesia, MAMC, New Delhi; 4 Professor, Department of Anaesthesia, MAMC, New Delhi; 5 Junior specialist, Government of NCT, Delhi; 6 Specialist Anaesthesia, Safdarjung Hospital, New Delhi, Department of Anaesthesia and Intensive Care, Maulana Azad Medical College & Associated Lok Nayak Hospital, New Delhi-110002

**Keywords:** Neuromuscular block, Temperature, Atracurium besylate, Vecuronium bromide, Amino acids

## Abstract

**Summary:**

Hypothermia is a common occurrence under anaesthesia and may prolong the duration of action of neuromuscular blockade. By limiting fall in temperature, an infusion of amino acid enriched solution may speed the recovery from neuromuscular blockade. We studied 60 ASA Grade – I/II patients of aged between 20 to 60 years scheduled for elective surgery under general anaesthesia. The patients were randomly divided into four groups to receive amino acid infusion with vecuronium bromide AV, normal saline with vecuronium bromide(CV), amino acid with atracurium besylate(A-At) and normal saline with atracurium besylate(C-At). Although there was a significantly lesser decrease in the core temperature from the baseline in all the patients receiving amino acid infusion (*p*<0.05), it significantly reduced the time to 25% recovery from the time of injection of vecuronium only. (60.59 ± 11.39 in CV vs 51 ± 14.72 min in AV) (P < 0.05), and not for atracurium.

## Introduction

Neuromuscular relaxants are integral part of modern anaesthesia practice. The duration of action is modified by various drugs (beta blockers^1^, calcium channel blockers^2^ etc), patient factors like age, hepatic^3^,^4^ or renal disease^5^, acid base^6^ and electrolyte imbalance. Recovery from neuromuscular blockade is also delayed in hypothermia^7^. The duration of action of vecuronium is doubled at 34 °C Vs 37 °C and duration of neuromuscular block produced by atracurium besylate is prolonged by 60% after 3 °C fall in core temperature.^3^

Administered nutrients (carbohydrates, fats and proteins) are digested and metabolized to produce energy in form of ATPs.^8^ However, a large portion of this energy becomes heat. Amino acids are known to increase the metabolic rate by 30 kcal for every 100 kcal provided by them, in contrast to 6 kcal and 4 kcal for carbohydrate and fat respectively.^9^ Infusion of amino acid enriched solution is reported to attenuate decrease in body temperature that occurs during general anaesthesia^10^–^12^ It has been shown that infusion of amino acids significantly hastens the recovery from neuromuscular blockade following vecuronium bromide.^13^

We hypothesized that infusion of amino acid enriched solution will also speed the recovery from neuromuscular blockade produced by atracurium bromide which is dependent on temperature sensitive Hoffmann's degradation for its metabolism. We designed this study to evaluate the effect of amino acid enriched solution on recovery from neuromuscular blockade caused by vecuronium bromide and atracurium besylate.

## Methods

After the Institutional Review Board approval and written informed consent, this prospective, randomized, double blind study was conducted on 60 ASA Grade I/II patients of either sex in the age group of 20-60 years admitted for elective surgery under general anaesthesia. Patients having known hepatic, neuromuscular disease, thyroid or renal disease, known allergy to study drug, receiving any drug known to affect neuromuscular transmission and BMI > 30 kg/ m^2^ were excluded from the study. The patients were randomly divided into four groups of 15 each to receive infusion of study drugs as follows:

Group AV: Amino acid infusion and vecuronium bromide.

Group CV: Normal saline and vecuronium bromide.

Group AAt: Amino acid infusion and Atracurium besylate.

Group CAt: Normal saline and atracurium besylate.

Patients were premedicated with IV fentanyl 2mcg.kg^−1^, 5 min before induction. All patients were induced with thiopentone sodium 5mg.kg^−1^ body weight. Depending upon the group allocation, vecuronium bromide (0.1mg.kg^−1^) or atracurium besylate (0.5mg.kg^−1^) were administered in a rapidly running intravenous infusion, and the airway was secured with a cuffed endotracheal tube of appropriate size at TOF 0 or at 5 min after neuromuscular blocking agent. Anaesthesia was maintained with nitrous oxide 66% in oxygen and 0.8% isoflurane. Ventilation was controlled to maintain normocapnia. The amino acid mixture used in our study was 10% w/v mixture of 20 amino acids (Celemin),

## Composition of Celemin

**Table:** 

Composition of Amino Acids (10% w/v and electrolytes) Each 100 ml contains:	
L - Isoleucine	0.510 g.
L - leucine	0.890 g.
L -Lysine Hydroclorided	0.700 g.
L - Methionine	0.380 g.
L - Phenylalanine	0.510 g.
L - Threonine	0.410 g.
L - Tryptophan	0.180 g.
L - Valine	0.480 g.
L - Arginine	-
Monohydrochloride	9.20 g.
L - Arginine	-
L - Histidine	0.520 g.
Aminoacetic Acid	0.790 g.
L - Alanine	1.370 g.
L - Glutamic Acid	0.460 g.
L - Aspartic Acid	0.130 g.
L - Proline	0.890 g.
L - Serine	0.240 g.
L - Tyrosine	0.030 g.
L - Cystine	-
L - Asparagine HO	0.372 g.
L - Cysteine	
Hydrochloride HO	0.073 g.
N - Acetyle-L-Tyrosine	0.123 g.
L-Ornithine Hydrochloride	0.320 g.
Total Amino acids	10.000 g.
Sorbitol	-
L-MalicAcid	0.100 g.
Sodium Acetate 3HO	0.395 g.
Potassium Acetate	0.245 g.
Magnesium Acetate 4HO	0.056 g.
Sodium Dihydrogen	
Phosphate 2HO	0.140 g.
Sodium Hydroxide	0.020 g.
Water for Injection	q.s.
**Electrolytes**	**mmol/L**.
Na^+^	45.00
K^+^	25.00
Mg^+^	2.50
Acetate	59.00
Cl-	62.00
H_2_PO_4_-	9.00
L-malate^2^-	7.50
Total Nitrogen	16.00 g/L
Non-protein Energy Content	-
Total Energy content	400 Kcal/L.
EAA: NEAA ratio	0.69
Osmolarity (mOsm/L.)	1040

Amino acid solution or normal saline was prepared in a micro drip set, by a person not further involved in the study and was handed over to the operator. A continuous intravenous infusion of the solution thus handed over was started at the rate of 100ml/h through a separate dedicated IV line before surgical incision and continued till reversal of the patient from anaesthesia. Ringer's lactate at operating room temperature was given for fluid replacement intraoperatively.

Datex Ohmeda AS3 multiparameter monitor was used for intraoperative monitoring. Temperature probe of the monitor was hung freely for one minute to record ambient temperature. Core temperature was monitored from a probe inserted in the nasopharynx and surface temperature was measured over adductor pollicis muscle. Temperature at the two sites (core and surface) was recorded before induction (pre induction), after securing the airway (baseline) and every 10 minutes thereafter.

Neuromuscular monitoring was carried out with TOF stimulation using a *TOF Watch*. The time from the injection of muscle relaxant to the return of the first, second, third and fourth response in TOF (T1, T2, T3, and T4) and time to 25% recovery of neuromuscular blockade was recorded and compared between the four groups.

At the end of surgery the residual neuromuscular blockade was reversed with atropine 0.02mg.kg^−1^ and neostigmine 0.06 mg.kg^−1^.

## Statistical analysis

We chose the group size of 15 each without performing power analysis because:

There was no study to go by where effect of amino acid on recovery from neuromuscular blockade caused by atracurium. The literature on vecuronium bromide was also limited.

After completing the study in the vecuronium group with N= 15 and α = 0.05 the power of study was found to be 75% for the vecuronium groups. The power couldnot be calculated for the atracurium as no difference was found in the test and control groups(AAt & CAt)

All the results are expressed as a mean ± standard deviation. Patient characteristics were compared using chi-square test. All the other data were compared using Student's t test.

## Results

The four groups were similar in their demographic profile, type and duration of surgery, ambient temperature and amount of fluids infused (Table 1) (*p*>0.05).

**Table 1:** Table showing patient, surgical procedure, study drug, fluids and OT related factors.

	VECURONIUM		ATRACURIUM	
Characteristic	CV(N = 15)	AV(N= 15)	CAt(N= 15)	AAt(N= 15)
• Sex (M/F)(n)	3/11	3/12	3/12	1/14
• Age (yrs.)				
Mean (SD)	41.18 (3.32)	36.75(9.66)	33.53(12.61)	34.27(9.50)
Range	21-60	23-50	18-58	21-55
• BMI (kg/m^2^)				
Mean (SD)	23.95 (3.32)	24.18 (3.19)	24.4 (2.82)	24.95 (3.56)
Range	20-30	17.80-29.60	21-28.90	18-29.35
• Surgical procedure				
Laparoscopic/ Non Laparoscopic$(n)	6/9	6/9	8/7	7/8
• Ambient temperature	26.1(2.0)	26(2.1)	25.5(2.4)	25.9(1.3)
• Duration of surgery(in minutes)	95.5 (46)	87(38)	91(52.4)	91.6(48.7)
• Volume of study drug (ml)	200(80)	186.2(51.2)	196.7(91.5)	180.3(76)
• Total fluid (ml)	1570(474)	1468.7(474)	1583.3(901)	1557(1241)

Values are mean (SD). C = control group, AA = amino acid group, BMI = body mass index

$ includes lower abdominal, retro peritoneal and upper abdominal surgeries. * P < 0.05

The time from injection of vecuronium bromide to return of T1, T2, T3, T4 were longer in the control group as compared to aminoacid groups, (p>0.05). The time to 25% recovery (T4/T1 25%) was 60.59 ± 11.39 minutes and 51 ± 14.72 minutes in the control and amino acid groups respectively (*p*<0.05).

The time to recovery from atracurium injection to return of T1, T2, T3, T4 and T4/T1 25% was similar in both the control and amino acid groups (Table 2) (*p*>0.05).

**Table 2:** Time to return of T1, T2, T3, T4 and T4/T1 25%

	VECURONIUM		ATRACURIUM	
NM recovery parameters (in mins.)	CV(N = 15)	AV(N= 15)	CAt(N= 15)	AAt(N= 15)
**T1**	29.38(9.14)	27.19(8.94)	27.8(8.49)	30.07(5.560
**T2**	36.47(9.50)	34.31(11.47)	35.36(7.73)	37.2(5.92)
**T3**	41.12(10.31)	39.38(12.32)	38.79(7.84)	41.07(6.69)
**T4**	44.47(10.61)	39.13(15.8)	39.79(8.52)	42.47(6.25)
**T4/T1 25%**	60.59(11.39)	51.0(14.72)*	49.87(7.90)	50.73(8.8)

Values are mean (SD). C = control group, AA = amino acid group NM = neuromuscular and N = number of patients.

* *P*<*0.05*

**Fig 1:**
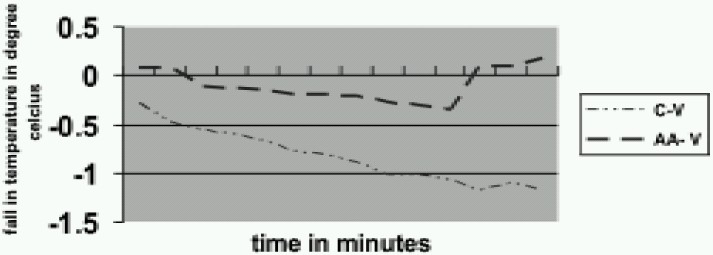
Changes in core temperature in vecuronium group

**Fig 2:**
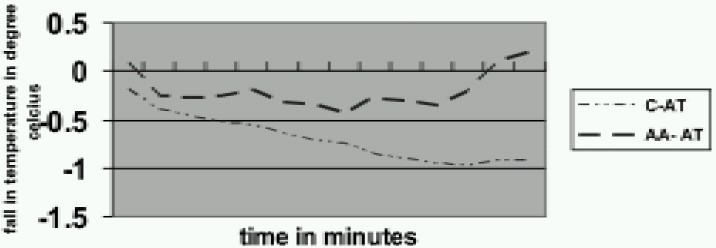
Changes in core temperature in atracurium group

Baseline core temperature was comparable in the four groups (*p*>*0.05*). The progressive decrease in core temperature (Dtc) was significantly more in the control groups (CV and CAt) than the aminoacid groups (AV and AAt) at all times of comparison (*p*<*0.05*). (Fig 1 & 2). In our study the greatest degree (50%) of fall occurred in the first 30 minutes (0.5 ±0.2 °C in CAt group and 0.6 ±0.3 °C in CV group). Infusion of amino acids prevented the fall in temperature in both the groups (1.0±0.6 °C in CV vs 0.4± 0.8 °C in AV, 0.96 ±0.3 °C in CAt vs 0.5 ± 0.3°C in AAt).

## Discussion

Our study supports that the infusion of amino acid solution hastens the recovery from vecuronium bromide induced neuromuscular blockade in anaesthetized patients while the recovery from the injection of atracurium besylate was not affected.

Core hypothermia impairs hepatic and renal metabolic function, so drugs like neuromuscular blocking agents may accumulate.^3^,^5^ In addition, hypothermia prolongs the duration of action of muscle relaxants by decreasing nerve conduction time, delaying repolarisation of nerve spike potential, slowing rate of acetylcholine release, decreasing receptor affinity and reducing rate of muscle contraction.^14^

Induction of anaesthesia is universally associated with hypothermia due to loss of normal thermoregulatory mechanism. This rapid fall in core temperature during first 40 minutes is followed by a gradual and constant decline in core temperature.^15^,^16^ In our study also the greatest degree (50%) of fall occurred in the first 30 minutes (0.5 ±0.2 °C in CAt group and 0.6 ±0.3 °C in CV group).

In our study the time from injection of vecuronium bromide to return of T1, T2, T3, and T4 were longer in the control group as compared to the amino acid group (Table 2) (*p*> *0.05*). The time to 25% recovery (T4/T1,25%) was significantly shorter in aminoacid groups as compared to control groups. The results are in line with previous study by Satioh et al showed that recovery following vecuronium bromide was significantly hastened by amino acid infusion (TOF T4/T1 120 minutes after administration of vecuronium, was 88% in AA group and 61% in the control group).^13^ Similarly the times from injection of vecuronium to return of the PTC 1 (Post tetanic count), T1, T2, T3 and T4 were shortened.^13^

In our study, the time from injection of atracurium besylate to return of T1, T2, T3, T4 and T4/T1 25% were similar in the control and the amino acid group (Table 2) (*p*>*0.05*). Amino acid infusion attenuated the fall in temperature in these patients but did not hasten the recovery from atracurium besylate (Table 2). This may be because unlike vecuronium bromide (85%-eliminated by liver), its elimination is independent of hepatorenal clearance, which gets deranged by hypothermia. Ester hydrolysis is responsible for two –third (67%) of elimination of atracurium which is not as temperature sensitive as metabolism of vecuronium bromide.^17^ Previous studies have also reported that 3°C decrease in core temperature doubles the duration of action of vecuronium bromide whereas the duration of action of atracurium increased by 60% only.^7^

It has been estimated that anaesthesia induced post-operative hypothermia and delayed recovery from neuromuscular blockade is a considerable clinical problem. Present findings may become clinically significant especially in patients who are prone to delayed neuromuscular recovery because of hypothermia like those in extremes of age^18^, undergoing prolonged surgeries^18^ and surgeries involving major fluid shifts^19^ (e.g. TURP, major bowel surgery) and surgeries in operating room with cooler ambient temperature (OT with laminar flow).^19^

Infusion of amino acid solution may be hazardous in patients with hepatic or renal disease, metabolic acidosis, disturbed amino acid metabolism and congestive heart failure. Hence, it should be avoided in such patients. It should also be avoided in children until tolerance studies have been performed.

This study clearly reaffirms that intraoperative infusion of amino acids speeds the recovery from vecuronium induced neuromuscular blockade in anaesthetized patients. However, similar effect could not be demonstrated for the recovery of atracurium.

## References

[1] Sessler DI (2000). Perioperative heat balance. Anesthesiology.

[2] Matsukawa T, Sessler DI, Sessler M (1995). Heat flow and distribution during induction of general anesthesia. Anesthesiology.

[3] Thorn berry EA, Mazumdar B (1988). The effect of changes in arm temperature on neuromuscular monitoring in the presence of atracurium blockade. Anaesthesia.

[4] Lebrault C, Berger JL, D' Hollander AA (1985). Pharmacokinetic and pharmacodynamics of vecuronium in patients with cirrhosis. Anesthesiology.

[5] Hunter JM, Jones RS, Utting JE (1984). Comparison of vecuronium, atracurium and tubocurarine in normal patients and in patients with no renal function. Br J Anaesth.

[6] Harrah MD, Way WL, Katzung BG (1970). The interaction of d-tubocurarine with antiarrythmic drugs. Anesthesiology.

[7] Bikhazi GB, Leung I, Foldes FF (1983). Calcium channel blockers increase potency of neuromuscular blocking agents in vivo. Anesthesiology.

[8] Lien CA, Matteteo RS, Ornstein E (1991). Distribution, elimination and action of vecuronium in the elderly. Anesth Analg.

[9] Feldman SA (1963). Effects of changes in electrolytes, hydration and pH upon the reactions to muscle relaxants. Br J Anaesth.

[10] Thorn berry EA, Mazumdar B (1988). The effect of changes in arm temperature on neuromuscular monitoring in the presence of atracurium blockade. Anaesthesia.

[11] Leslie K, Sessler DI, Bjorksten A R, Moayeri A (1995). Mild hypothermia alters propofol pharmacokinetics and increases the duration of action of atracurium. Anesth Analg.

[12] Sellden E, Branstrom R, Brundin T (1996). Augmented thermic effect of amino acids under general anesthesia occurs predominantly in extra splanchnic tissues. Clin Sci.

[13] Sellden E, Branstrom R, Brundin T (1996). Preoperative infusion amino acids prevents postoperative hypothermia. Br J Anaesth.

[14] Sellden E, Brundin T, Wahren J (1994). Augmented thermic effect of amino acids under general anesthesia: a mechanism useful for prevention of anesthesia induced hypothermia. Clin Sci.

[15] Guyton AC, Hall JE (2000). Energetics and metabolic rate. Text-book of Medical Physiology. WB Saunders.

[16] Ganong WF (1995). Energy balance, Metabolism and Nutrition. Review of Medical Physiology. Appleton and Lange.

[17] Satioh Y, Kaneda K, ToKunaga Y, Murakawa M (2001). Infusion of amino acid enriched solution hastens the recovery from neuromuscular blockade cause by vecuronium. Br J Anaesth.

[18] Merrett RA, Thompson CW Webb FW (1983). In vitro degradation of atracurium in human plasma. Br J Anaesth.

[19] Kongsayreepong S, Chaibundit C, Chadpaibool J (2003). Predictor of core hypothermia and surgical intensive care unit. Anesth Analg.

[20] Sessler DI, Miller RD (2000). Temperature monitoring. Anesthesia.

